# Multifaceted regulation of the *HOX* cluster and its implications in oral cancer

**DOI:** 10.1186/s13148-025-01933-w

**Published:** 2025-07-17

**Authors:** Kanaka Sai Ram Padam, Keith D. Hunter, Raghu Radhakrishnan

**Affiliations:** 1 Department of Cell and Molecular Biology, Manipal School of Life Sciences, Manipal Academy of Higher Education, Manipal, Karnataka 576104 India; 2 Liverpool Head and Neck Centre, Molecular and Clinical Cancer Medicine, University of Liverpool, Liverpool, UK; 3 Department of Oral Pathology, Manipal College of Dental Sciences, Manipal, Manipal Academy of Higher Education, Manipal, 576104 India; 4 Academic Unit of Oral and Maxillofacial Medicine and Pathology, School of Clinical Dentistry, University of Sheffield, Sheffield, S10 2TA UK; 5 Unit of Oral Biology and Oral Pathology, Oman Dental College, Muscat 116, Oman

**Keywords:** Epigenetics, *HOXB9*, Methylation, Transcriptome, Antisense

## Abstract

**Background:**

The hypothesis that aberrant expression of homeobox (HOX) transcription factors contributes to oral cancer progression is gaining prominence. However, the mechanism of regulation involved in the clustered dysregulation of *HOX* clusters is not clearly known.

**Results:**

Our findings revealed that *HOXA* and *HOXB* clusters showed significant locus-specific CpG methylation changes compared with the *HOXC* and *HOXD* clusters. The constitutively unmethylated regions identified in the *HOXA1*, *HOXA11*, *HOXB5*, *HOXB6*, *HOXB9*, *HOXC5*, *HOXC10* and *HOXC11* genes may be associated with open chromatin-mediated gene regulation. The methylation of CpG loci within the intron of *HOXB9* may serve as a potential marker for distinguishing patients with premalignant and advanced oral tumors. *HOXA5* and *HOXC9* showed higher transcription factor-mediated interactions with neighboring *HOX* genes within and across the clusters. Additionally, *HOXB9* and *HOXC10* were predicted to directly regulate the G2–M checkpoint and hypoxia pathways. *HOXA* genes can be post-transcriptionally regulated through an antisense-mediated mechanism involving embedded *HOX* long noncoding RNAs (lncRNAs). Posterior *HOX* genes were more highly expressed than anterior *HOX* genes. The *HOXC* and *HOXD* cluster gene expression patterns were similar to those of the embedded lncRNAs. *HOXA1*, *HOXC13* and *HOXD10* were significantly correlated with the cancer hallmarks driving oral carcinogenesis.

**Conclusion:**

The functional consequence of *HOX* genes dysregulation was driven by diverse DNA and RNA epigenetic mechanisms affecting the transcriptional and post-transcriptional regulation contributing to the oral cancer progression.

**Supplementary Information:**

The online version contains supplementary material available at 10.1186/s13148-025-01933-w.

## Background

With over 377,713 new cases and 177,757 deaths reported in 2020, oral cancer affecting the lips, tongue, gums, floor of the mouth, palate and other mouth areas (ICD 10—C00–C06) has been ranked the 13th most common cancer globally [1]. The occurrence of oral squamous cell carcinoma (OSCC) is a stagewise process [2] that results from a breakdown in genomic integrity due to continued exposure to tobacco-related carcinogens [3] and HPV infection [4]. Oral premalignant lesions are a diverse group of clinical entities that have an unpredictable risk of malignant transformation [5] through their varying morphological alterations [6]. Molecular studies have revealed identical genetic changes in precancerous and cancerous oral lesions from the same patient, implying that the progeny of the cells in a dysplastic oral lesion may eventually result in malignancy [7, 8]. Timely detection of oral cancer in its earliest stage of development has improved cure rates and quality of life.

Oral cancer is a complex disease driven by genetic and epigenetic alterations that disrupt normal cellular processes, leading to uncontrolled tumor growth. Many critical cancer-associated genes regulate cancer hallmarks. The high frequency of mutations in tumor protein 53 (TP53) dysregulates cell-cycle checkpoints and suppresses apoptosis [9], whereas dysregulation of PIK3CA (phosphatidylinositol-4,5-bisphosphate 3-kinase catalytic subunit alpha) activates the PI3K/Akt pathway, leading to therapeutic resistance and tumor progression [10]. Additionally, CCND1 (Cyclin D1), a key cell-cycle regulator, is often overexpressed in oral cancer, driving uncontrolled cell proliferation and tumor development [11]. These genetic alterations underpin fundamental cancer hallmarks in oral cancer, highlighting the complexity of its molecular landscape.

*HOX* genes belong to a superfamily of evolutionarily conserved genes encoding transcription factors essential for early development, morphogenesis and maintenance of cellular identity. In mammals, a total of 39 *HOX* genes are organized into four clusters: *HOXA*, *HOXB*, *HOXC* and *HOXD.* The homeobox coding sequence within homeotic genes, known as the homeodomain, was the elemental DNA binding motif in *HOX* gene family [12]. Aberrant expression of *HOX* genes disrupts normal developmental processes and promotes sustained proliferation, evasion of apoptosis, and metastasis [12, 13]. Recent studies [14, 15] have revealed that dysregulated *HOXs* act as transcription factors and affect the cell cycle, epithelial-to-mesenchymal transition, invasion and angiogenesis. However, the regulation of *HOX* clusters and their role in disease development remain poorly understood.

Dysregulation of *HOX* genes in oral cancer is gaining prominence, but the epigenetic landscape of the *HOX* cluster coordinating the clustered expression and intricate multifaceted regulatory mechanisms contributing to transcriptional misregulation in cancer remains poorly understood. This study focused on understanding the regulation of the *HOX* cluster and its implications in OSCC.

## Materials and methods

### Gene sequence retrieval and mapping

Homeobox and embedded noncoding RNA (ncRNA) gene sequences, along with 1 kb of upstream nucleotides relative to the annotated transcription start site (5′ end) of the gene, were retrieved from the University of California, Santa Cruz (UCSC) [16] genome browser and mapped using the National Center for Biotechnology Information Reference Sequence (NCBI—RefSeq) database. The alternative transcripts of the embedded ncRNAs were ordered from the 5′ to 3′ end, following the exon‒exon structure. Additionally, the query was explored via UniProtKB (https://www.uniprot.org/) to retrieve the experimentally validated HOX protein sequence. The retrieved protein sequences were aligned with their corresponding nucleotide sequences from GenBank via ExPASy (https://web.expasy.org/translate/) to map homeobox and intergenic regions on the basis of GenBank annotations. The natural antisense properties of the embedded lncRNAs within the *HOX* cluster were screened, highlighting the targeted exons of *HOX* genes.

### Clinical specimen collection

The study included matched potentially malignant oral lesions (*n* = 15) (aged 37–81 years, median—62.67 years), and 32 oral cancer samples and 30 adjacent matched normal tissue samples (aged 37–79 years, median—56.17 years) were collected from patients undergoing surgery at Kasturba Medical College (KMC), Manipal, India, with informed consent from Institutional Ethics Committee (IEC: 348/2018). Patients who had undergone prior radiotherapy or chemotherapy as well as HPV-positive cases were excluded. Data on risk factors, including tobacco use, alcohol consumption, and areca nut chewing, were inconsistently documented across patients and were therefore excluded from the analysis to maintain data integrity. The samples were categorized into (a) potentially malignant oral lesions—PMOLs (*n* = 15), (b) oral squamous cell carcinoma (OSCC, *n* = 32), (c) locoregional tumors without lymph node involvement comprising stages I and II (TN0, *n* = 15), and (d) invasive locoregional tumors with or without node involvement comprising stages III and IV (TN0 +, *n* = 17). The clinicopathologic details are provided in Table 1.

**Table 1:** Clinicopathologic profile of samples collected in the present study (IEC: 348/2018)

Group	Sample size (n)	Median age	Pathological staging			Site(s)
			T	N	M	
Normal	30	56.17	–	–	–	Adjacent tissue specimen
PMOL	15	62.67	–	–	–	Dysplastic oral lesions of buccal mucosa (*n* = 4), tongue (*n* = 5), alveolus (*n* = 3) and gingiva (*n* = 3)
OSCC	32	56.71	T1	N0	M0	Alveolus (*n* = 2), Tongue (*n* = 6), Buccal Mucosa (*n* = 19), Floor of the mouth (*n* = 5)
			T2	N1		
			T3	N2		
			T4	N3		
TN0	15	60.53	T1	N0	M0	Alveolus (*n* = 2), Tongue (*n* = 2), Buccal mucosa (*n* = 9), Floor of mouth (*n* = 2)
			T2			
TN0 +	17	53.35	T3	N0	M0	Tongue (*n* = 4), Buccal mucosa (*n* = 10), Floor of mouth (*n* = 3)
			T4	N1		
				N2		
				N3		

### Methyl-capture sequencing

Gene-wide methylation and locus-specific CpG methylation patterns were assessed in a panel of PMOL (*n* = 8), TN0 (*n* = 6) and TN0 + (*n* = 8) samples by performing methyl-capture sequencing (MC-seq). Library preparation was performed using the Illumina-compatible SureSelectXT methyl-seq target enrichment (Agilent Technologies), and 500 ng of isolated genomic DNA was sheared to generate fragments (150–200 bp) via a Covaris S2 sonicator (Covaris). End-repair, adenylation and ligation to adapters were followed by enrichment and hybridization using SureSelectXT Human methyl-seq probe (Agilent Technologies). The enriched and purified library underwent bisulfite conversion using the EZ DNA methylation Gold kit (ZymoResearch). Bisulfite-converted DNA was amplified (8 PCR cycles), followed by PCR indexing amplification (6 PCR cycles). The libraries were paired-end sequenced for 150 cycles on an Illumina HiSeq X Ten sequencer (Genotypic Technology Pvt. Ltd., India). The raw reads were quality-checked using FastQC v0.11.3 [17] tool. High-quality processed reads (> Q30) were obtained using Trim Galore (v0.4.0, Babraham Bioinformatics) and aligned using Bismark [18] against the hg19 genome. The alignments were used for Bismark methylation extraction, mapping the extracted CpG contexts to homeobox genes from the promoter to the gene body. The *HOX* gene regions (1 kb upstream and 100 bp downstream of the TSS as putative promoters) were screened to identify the constitutively unmethylated regions (CURs) associated with a loss of methylation compared with adjacent loci, which was consistent across all sample types. We employed a stringent cut-off o*f* < 10% variation in CpG methylation within and between the case‒control samples to determine the constitutively unmethylated regions (CURs). The average CpG-specific methylation across the *HOX* genes in each group was analyzed using the ggplot2 R package. A heatmap of *HOX* gene region-specific methylation percentages was created using the pheatmap R package, wit*h* > 25% considered hypermethylated an*d* < 25% considered hypomethylated.

### ROC-AUC analysis

Receiver operating characteristic-area under the curve (ROC-AUC) analysis using pROC and randomForest R packages was used to predict altered *HOXB9* intron CpG methylation as a biomarker to distinguish between premalignant and advanced oral cancer. The logistic regression model was performed with diseased cases as a test set and normal tissue as a control set to determine the ROC curve using false-positive (1-specificity) and true-positive (sensitivity) rates. Model stringency was assessed by constructing a random forest model, and the area under the curve (AUC) was used to determine the 95% confidence interval.

### Chromatin accessibility of *HOX* genes

The Genomic Data Commons (GDC) pan-cancer cohorts were accessed to retrieve ATAC-seq (Assay for transposase-accessible chromatin) (*n* = 404) [19], and whole-genome bisulfite sequencing (WGBS) datasets (*n* = 8 normal and *n* = 39 primary tumors) from the University of California, Santa Cruz (UCSC) Xena browser [20], covering 23 cancer types from The Cancer Genome Atlas Program (TCGA). Tumor type-specific stratification of ATAC-seq data was subsequently performed to investigate patterns of chromatin accessibility at genomic regions of interest. This approach enabled the identification of cancer-specific regulatory trends, particularly at loci exhibiting constitutive unmethylation, thereby providing insights into the potential epigenetic basis of chromatin remodeling across diverse tumor types. The data were analyzed and visualized using R programming (R Core Team (2020)). R: A language and environment for statistical computing. R Foundation for Statistical Computing, Vienna, Austria. URL https://www.R-project.org/).

### Upstream promoter binding factors on *HOX* genes in the cluster

Chromatin-regulating factors on *HOX* promoters were analyzed using the TRANSFAC 2020.1, GeneXplain [21] Match™ tool, which predicts TF binding sites in *HOX* promoters using positional weight matrices. Furthermore, the Harmonizome [22] pipeline consists of 112 datasets and 65 databases, including ChEA [23], MotifMap [24], TRANSFAC [25], JASPAR [26] and ENCODE [27], which identify upstream targets that act on *HOX* genes.

### Retrieval of HOX transcription factor target profiles

The targets of HOX proteins were accessed and curated from TRED (Transcriptional Regulatory Element Database) [28], ITFP (Integrated Transcription Factor Platform) [29], and TRRUST (Transcription Regulatory Relationships Unraveled by Sentence-based Text-mining) [30], which provides mammalian transcription factor profiles, as well as the Interactome database [31], which includes the transcription factor-directed transcription factor interactions identified through DNase I footprinting. Duplicate entries were removed, and the data were compiled. The Harmonizome pipeline [22] was used to explore transcription factor databases such as ChIP-X enrichment analysis (ChEA) [23], MotifMap [24] and JASPAR [26] to identify HOX proteins that act on target gene promoters. Furthermore, the cancer hallmark gene sets summarizing the biological states or processes were retrieved using Molecular Signatures Database (MSigDB) collection [32].

### RNA‒protein interactions of HOX lncRNAs

HOX cluster-embedded long noncoding RNA (HOX lncRNA) interactions were predicted using the RNA Interactome (RNAInter v4.0) [33] and NPInter v5.0 [34] databases, which curate functional interactions between long noncoding RNAs and proteins sourced from peer-reviewed publications and high-throughput experimental studies, such as Capture Hybridization Analysis of RNA Targets (CHART-seq), Chromatin isolation by RNA Purification (ChIRP-seq), Cross-linking, Ligation and Sequencing of Hybrids (CLASH) and Cross-linking and Immunoprecipitation (CLIP-seq). The interactions of HOXlncRNAs with *HOX* genes and transcription regulators were visualized as a network using the Cytoscape software [35].

### Whole-transcriptome sequencing

A cohort comprising PMOL samples (*n* = 7 premalignant and adjacent normal samples) and OSCC samples (*n* = 15 normal; *n* = 18 tumor) was processed for whole transcriptome sequencing. Briefly, total RNA isolation was performed using mirVana™ miRNA Isolation kit (Cat. No. AM1560, Invitrogen). Library preparation was carried using NEBNext RNA ultra II (NEB #E7775, US). The rRNA content of cytoplasm and mitochondria was removed using biotinylated, target-specific oligos and rRNA removal beads. Followed by purification and the first-strand cDNA synthesis using random hexamers and second-strand cDNA synthesis by USER enzyme-based digestion to preserve the functional strand mapping to the coding strand. Enrichment and indexing were carried out using limited-cycle PCR, followed by AMPure bead purification to construct the cDNA libraries. The prepared libraries were sequenced on Illumina HiSeq4000/X system (MedGenome Labs Ltd., India) to generate 60 million, 2 × 150 bp paired-end reads per sample. Quality check (> Q30) and preprocessing of the raw data were carried out using Trimmomatic (v0.36) and Bowtie2 (v2.2.4), which were used to quality check (*Q* > 30) and pre-process the obtained raw reads. Data were aligned to the human reference genome (hg19) using HISAT2, and read counts mapped to genes were obtained via FeatureCounts.

### Differential expression analysis

The raw read counts from the PMOL, TN0, TN0 + and pooled OSCC tumor cohorts were corrected for batch effects, library preparation and confounding variations using between-sample upper quartile normalization [36] using RUVseq [37] in the R program. Differential gene expression (DGE) analysis was performed using the DESeq2 R package, setting a threshold of log2FC (≤ − 1.5 and ≥  + 1.5) with *p* < 0.05 to control for a 10% FDR. *HOX* genes and the embedded noncoding RNAs were visualized as heatmap using pheatmap R. Pearson correlation analysis revealed that the gene pair associations with *r* > 0.3 were moderate and those with *r* > 0.7 were considered strongly correlated, with *p* < 0.05 being statistically significant.

### Functional overrepresentation analysis

The downstream functional consequences of aberrantly regulated *HOX* genes were analyzed by accessing gene ontology (GO)-biological process (BP), molecular function (MF) and Kyoto Encyclopedia of Genes and Genomes (KEGG) [38] pathways using the ClusterProfiler [39] R package. For overrepresentation analysis, the subset of upstream factors acting on *HOX* genes and the downstream transcriptionally regulated target genes were compiled as a query set. An adjusted *p*-value (< 0.05) with the Bonferroni‒Hochberg correction was used to determine the statistical significance between the query set and the mapped hits.

## Results

### The *HOX* cluster is differentially methylated in OSCC

**Fig. 1:**
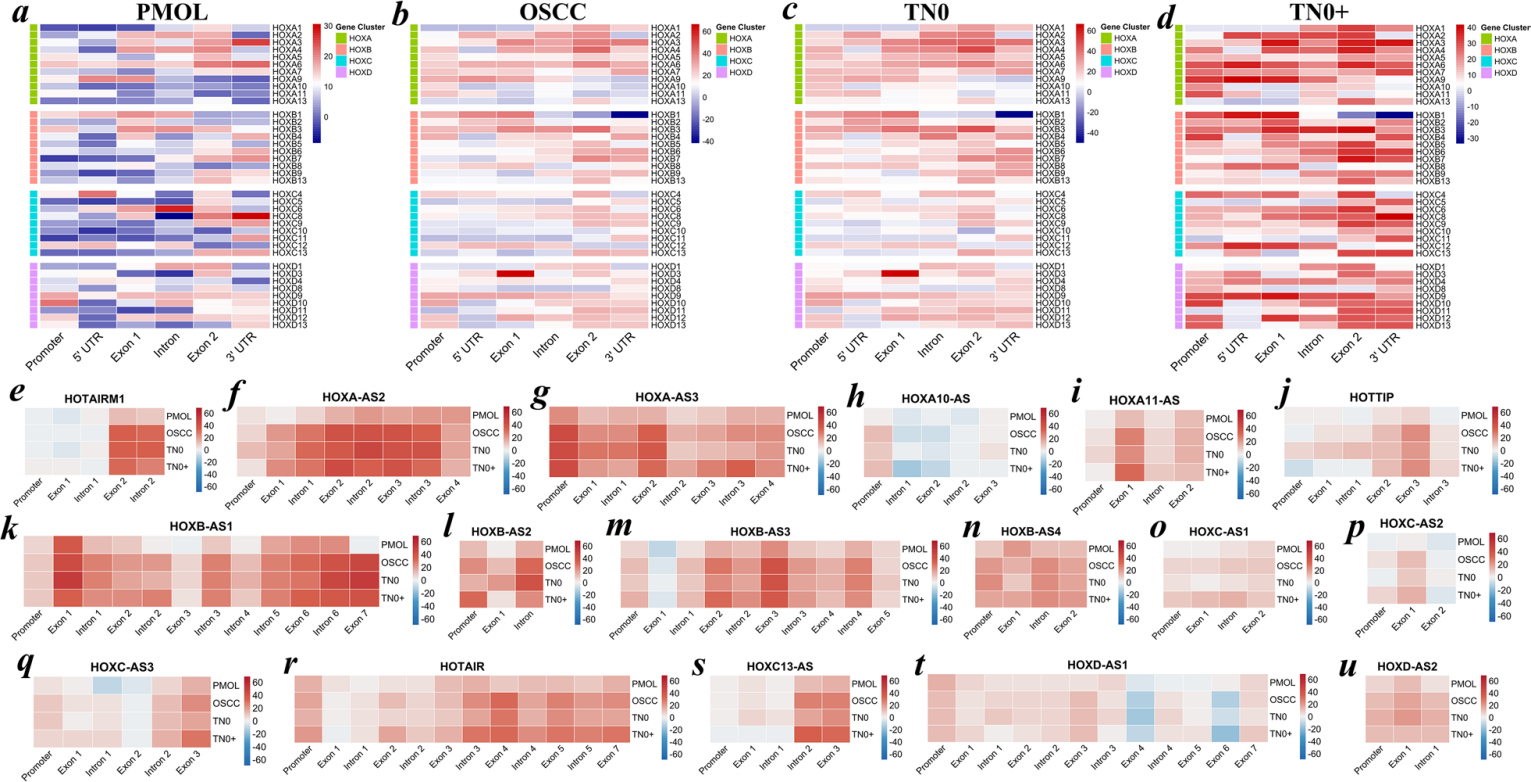
**a–u** Region-specific methylation of *HOX* genes during oral cancer progression. The methylation profile of homeobox genes in the HOX cluster was represented as a percentage of methylation differences across the regions in the **a** PMOL, **b** OSCC, **c** TN0 group of OSCC patients and **d** TN0 + group of OSCC patients compared with their respective matched normal cases. Further, the methylation difference of lncRNAs belonging to the **e–j** *HOXA* cluster, **k–n** *HOXB* cluster, **o–s** *HOXC* cluster and **t–u** *HOXD* were illustrated as a heatmap. A cut-of*f* > 25% was considered hypermethylated, and a cut-of*f* < −25% was considered hypomethylated. The methylation of the CGs was averaged across the region per gene and illustrated as a heatmap over a percentage of methylation difference compared to the pooled normal. PMOL—potentially malignant oral lesions; OSCC—oral squamous cell carcinoma; TN0—non-invasive OSCC; TN0 + —invasive OSCC; lncRNAs—long noncoding RNAs

Differential methylation patterns were observed in *HOX* clusters across the gene body, with a significant increase in the OSCC samples compared to the PMOLs. Specifically, exonic methylation of *HOXA4* and *HOXD3,* as well as intronic methylation of *HOXA6* and *HOXB9,* differed significantly between OSCC and PMOL samples. A progressive increase in promoter-to-exonic methylation was noted in the paralogous *HOXA9* and *HOXD9* genes with increasing tumor stage (Fig. 1a–d). Compared with premalignant cases, *HOX* cluster-embedded lncRNAs also exhibited differential methylation patterns in exonic regions in tumor cases. Notably, *HOXA-AS2*, *HOXA-AS3, HOXB-AS1* and *HOXB-AS3* were hypermethylated in tumors compared with PMOLs, whereas *HOTAIR* exhibited increased methylation in the gene body. *HOXB* cluster antisense lncRNAs, such as *HOXB-AS1*, *HOXB-AS2*, *HOXB-AS3* and *HOXB-AS4,* displayed variable gene body methylation patterns across tumor groups, in contrast to the *HOXC* and *HOXD* cluster lncRNAs. Notably, *HOXC* antisense lncRNAs such as *HOXC-AS1*, *HOXC-AS2*, *HOXC-AS3* and *HOXD-AS1* were unmethylated. *HOTAIRM1* and *HOXC13-AS* exhibited significantly differential methylation patterns across the gene (Fig. 1e–u). These varied gene body methylation patterns suggest diverse roles of DNA methylation in regulating *HOX* cluster expression. A schema illustrating the *HOX* genes, along with the embedded ncRNAs and their temporospatial positioning across the clusters, is shown in Additional file 1.

### *HOXB9* intronic CpG sites as a marker of diagnostic relevance

**Fig. 2:**
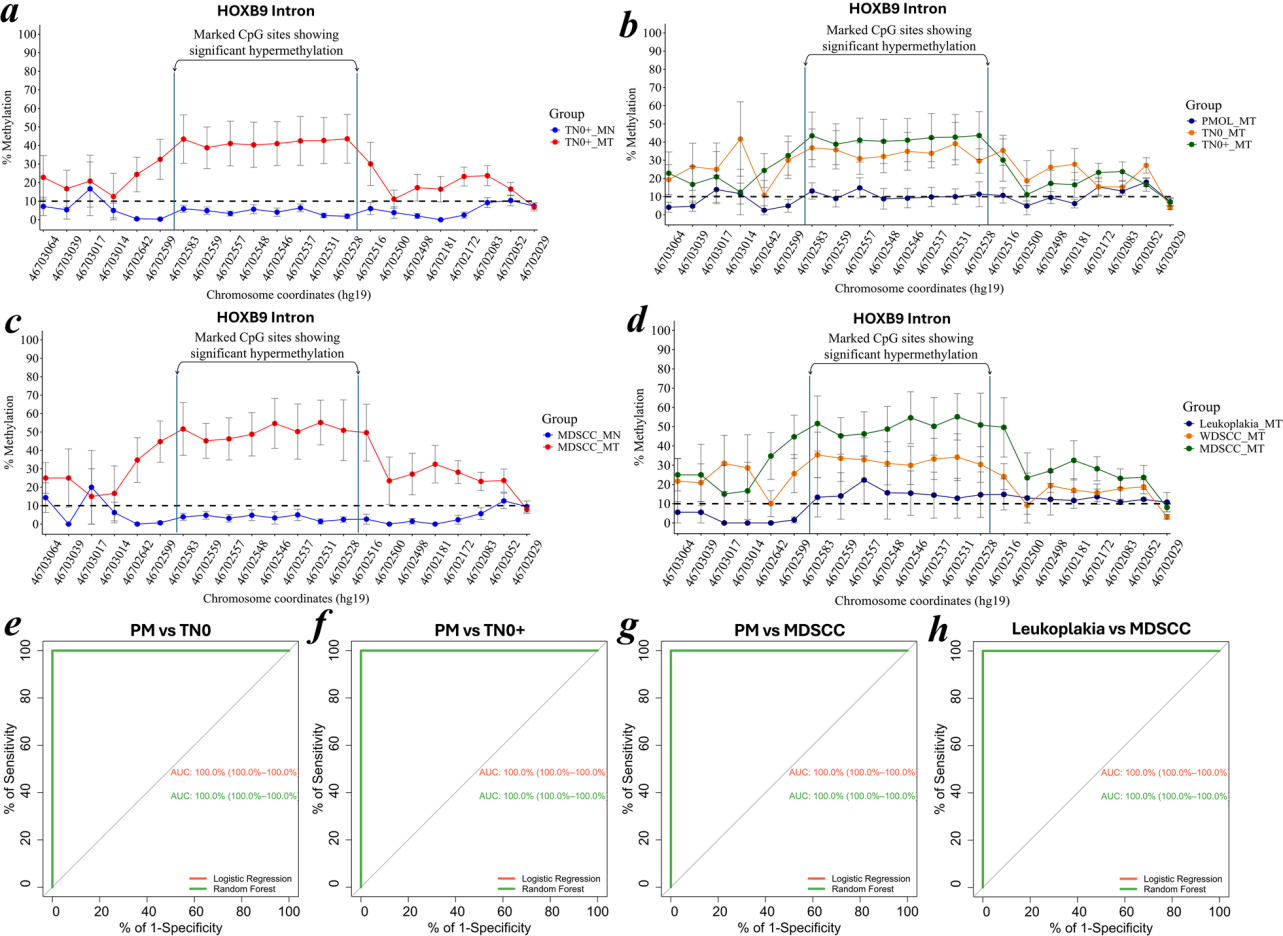
**a–h** Locus-specific CpG sites belonging to the intronic region of *HOXB9* were unmethylated in the PMOL and exhibited hypermethylation in the TN0 + group. **a-b** The 8 specific CpG sites (hg19/chr17:46702528–46702583:−1) exhibited consistent and significant hypermethylation in advanced TN0 + tumors (*n* = 8 matched cases) compared with the PMOL (*n* = 8 matched cases) and TN0 (*n* = 6 matched cases) cohorts. Furthermore, the categorization of the patient samples based on **c–d** leukoplakia (*n* = 3 matched cases), well-differentiated squamous cell carcinoma cases (*n* = 8 matched cases), and moderately differentiated squamous cell carcinoma (*n* = 5 matched cases) revealed a similar pattern of hypermethylation. The pooled locus-specific CpG-specific methylation data was presented as the mean with standard error normalized to the matched normal samples. **e–h** ROC-AUC analysis of locus-specific CpG methylation demonstrated predictability as a diagnostic biomarker to differentiate between the leukoplakia, TN0 and TN0 + stage groupings of OSCC. Statistical significance was defined by a confidence interval (CI) > 95%. MN refers to matched normal tissue and MT refers to matched tumor tissue, representing samples obtained from the same case

*Homeobox B9* gene-wide methylation patterns revealed significant variability in the CpG locus located in the intronic region (Additional file 2). The eight locus-specific CpG sites (hg19/chr17:46702528–46702583-1) showed significant hypermethylation in the advanced TN0 + group (*n* = 8 matched cases) compared to the PMOL (*n* = 8 matched cases) and TN0 (*n* = 6 matched cases) patient groups. The pattern was notably more evident in moderately differentiated squamous cell carcinoma (*n* = 5) patients than in well-differentiated patients (*n* = 8 matched patients) and leukoplakia patients (*n* = 3 matched patients) (Fig. 2a–d). The ROC curve demonstrated high sensitivity and specificity of the identified CpG markers in distinguishing leukoplakia from early and advanced tumors, with a confidence interva*l* > 95% (Fig. 2e–h). These results underscore the clinical relevance of CpG site-specific methylation changes in *HOXB9* during oral cancer progression.

### *HOX* genes constitute open chromatin regions in unmethylated regions

**Fig. 3:**
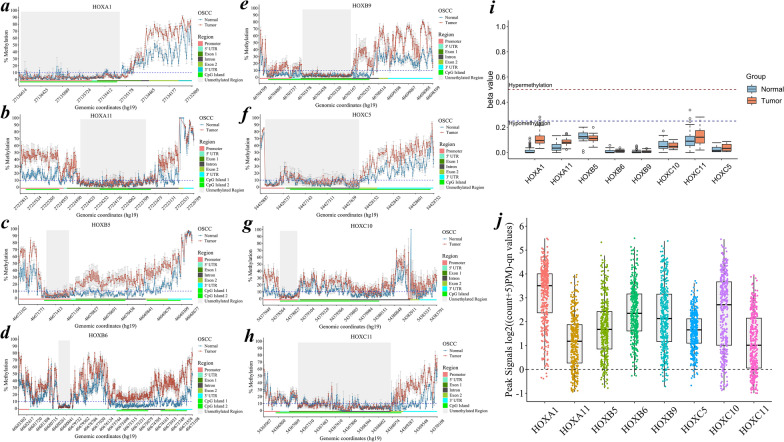
**a–j** Gene-wide methylation profile of *HOX* genes exhibiting CUR marks in case‒control (*n* = 14) samples independent of sample type. **a** *HOXA1* within the promoter and exon 1 (hg19/chr7:27135302–27136558-1), **b** *HOXA11* within the first exon with an overlap of introns (hg19/chr7:27223859–27224500:−1), **c** *HOXB5* nearest the TSS of the gene body (hg19/chr17:46671203–46671443:−1), **d** *HOXB6* in the 5′ UTR (hg19/chr17: 46679968–46680261:−1), **e** *HOXB9* within exon 1 (hg19/chr17:46703140–46703596:−1) which varied across the gene body. **f** *HOXC5* within the CpG sites nearest to the promoter (hg19/chr12:54426390–54426695:1) and **g** *HOXC10* displayed variable CUR marks (hg19/chr12:54378698–54378899:1) and **h,** *HOXC11* within the intronic region (hg19/chr12:54367152–54368797:1), in a consistent pattern compared with the CpG site signals in the adjacent loci. A stringent cut-off o*f* < 10% for within and between the samples of the site-specific CpG signal in the case‒control samples was used. The regions shaded in gray are the CUR marks screened in the present study. Furthermore, the exploratory analysis using public datasets revealed enriched peak signals in the CUR marks of the *HOX* genes in **i** WGBS cohort (*n* = 8 normal and *n* = 39 tumor samples) of the PanCan TCGA dataset and **j** ATAC-seq analysis of the PanCan dataset (*n* = 404). The data was presented as log2((coun*t* + 5)PM)-qn values). CUR—constitutively unmethylated regions; PanCan—pancancer; TCGA—The Cancer Genome Atlas; WGBS—whole-genome bisulfite sequencing; ATAC-seq—Assay for transposase-accessible chromatin sequencing

Gene-wide methylation screens of the *HOX* cluster revealed consistent patterns of constitutively unmethylated regions (CURs), characterized by methylation loss regardless of the cell type and disease state (Fig. 3a–h). These patterns are strongly associated with the segments of CpG islands and are usually located upstream of the gene body. Notably, our results revealed that *HOXA1*, *HOXA11*, *HOXB5*, *HOXB6*, *HOXB9*, *HOXC5*, *HOXC10* and *HOXC11* constitute these unmethylated regions. Among these genes, *HOXA1*, *HOXB5*, *HOXB6*, *HOXC5* and *HOXC10* presented CURs upstream of the gene body, whereas *HOXA11*, *HOXB9* and *HOXC11* presented these distinctive marks in exonic regions. Similar observations were noted in PMOLs exhibiting these CURs at *HOX* loci (Additional File 3), mirroring the patterns observed in the OSCC samples (Fig. 3a–h). Whole-genome bisulfite sequencing of the PanCan cohort revealed similar trends (Additional file 4) (Fig. 3i). ATAC-seq data from the PanCan cohort uncovered open chromatin signals in these unmethylated regions (Fig. 3j). Furthermore, tumor-type-specific analysis using ATAC-seq data showed distinct patterns of open chromatin signals likely corresponding to gene-specific expression signatures (Additional File 3). These findings suggest that CURs in the identified *HOX* genes are observed across multiple cancer types, implying that their regulation may be driven by chromatin accessibility in the disease state.

### Posterior *HOX* genes were aberrantly expressed during oral cancer progression

**Fig. 4:**
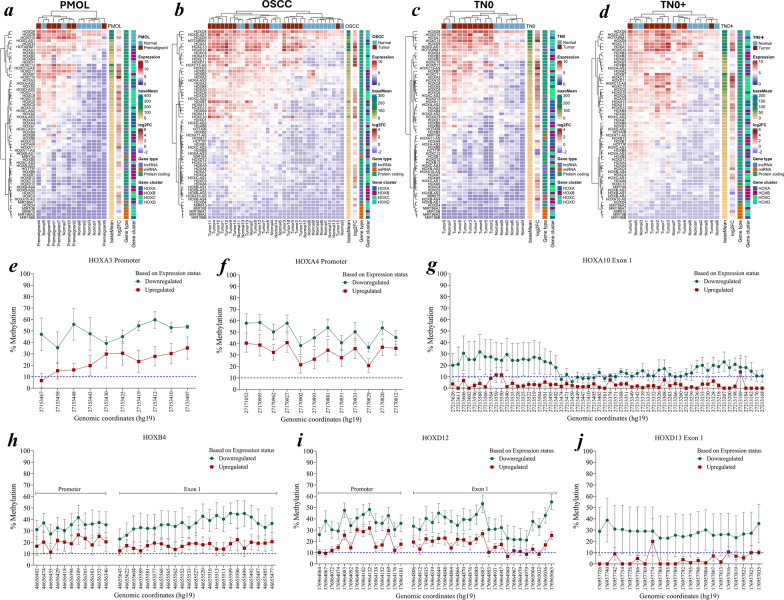
**a–j** Expression of *HOX* cluster and DNA methylation dynamics. DGE analysis of homeobox genes and embedded ncRNAs in the *HOX* cluster in a cohort of **a** PMOLs, **b** OSCC, **c** TN0 and **d** TN0 + stage groupings of OSCC. Promoter and exon methylation of **e** *HOXA3*, **f** *HOXA4*, **g** *HOXA10*, **h** *HOXB4*, **i** *HOXD12*, and **j** *HOXD13* in the patient cohort (*n* = 14) on the basis of the status of gene expression. A cut-of*f* < 10% (represented as dashed blue lines) was considered unmethylated. DGE—Differential gene expression; ncRNA—noncoding RNA; PMOL—potentially malignant oral lesions; OSCC—oral squamous cell carcinoma; TN0—non-invasive OSCC; TN0 + —invasive OSCC

Posterior *HOX* genes were significantly upregulated (logF*C* > 1.5, pad*j* < 0.05) in oral cancer samples compared with normal samples. The upregulation of *HOXA1*, *HOXA10*, *HOXA11*, *HOXB7*, *HOXC8*, *HOXC13* and *HOXD10*, *HOXC-AS1, HOXC-AS2* and *HOXC13-AS* was consistent across the TN0 and TN0 + groups. Furthermore, the categorization of samples based on tumor progression revealed that *HOXA3* was upregulated in the TN0 samples compared to TN0 + samples, indicating differential expression across the groups. The lncRNAs *HOTAIRM1*, *HOXC-AS1, HOXC-AS2* and *HOXC13-AS* were significantly upregulated in both the TN0 and TN0 + groups, whereas *HOXD-AS1* was upregulated only in the TN0 + group. Consistent upregulation of *HOXC* genes and embedded lncRNAs implicates their coregulatory role in carcinogenesis (Fig. 4a–d).

### Promoter- and exon-driven methylation regulates the expression of *HOX* genes

Based on the expression status, the categorization of patient samples (*n* = 14) that were common across the *HOX* methylome and transcriptome signatures revealed distinct locus-specific CpG methylation patterns. The downregulated *HOXA3* and *HOXA4* samples exhibited increased promoter methylation, whereas *HOXA10* first exon methylation was negatively correlated with the expression state. Furthermore, the promoter and first exon methylation of *HOXB4* and *HOXD12,* and the exonic methylation of *HOXD13* were inversely correlated with the expression (Fig. 4e–j). These results suggest that the methylation of the promoter and the first exonic region of *HOX* genes represses their expression. In contrast, a positive association between whole-gene methylation and expression was observed for *HOXA9* and *HOXD9.* Additionally, methylation of the second exon in *HOXA1*, *HOXB5*, *HOXC13* and *HOXD10* showed a positive correlation with gene expression (Additional file 5).

### Transcription factor-mediated regulation of the chromatin architecture and cancer hallmarks

**Fig. 5:**
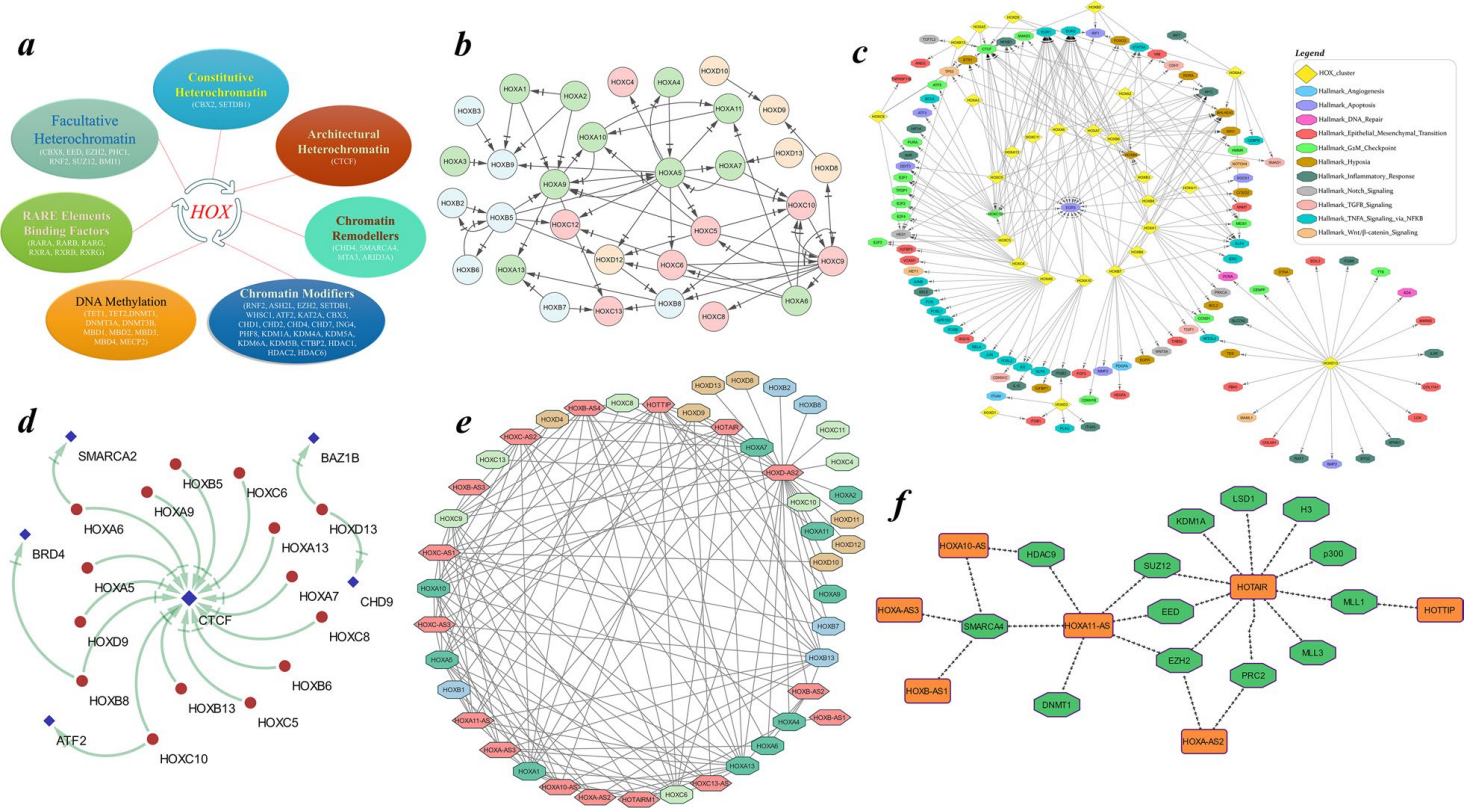
**a–f** Transcription factor-mediated epigenetic regulation of the *HOX* cluster. **a** Schematic illustration of the epigenetic factors that act on the promoters of homeobox genes. **b** Transcriptional regulation of HOX within and between neighboring clusters. **c** Downstream targeting of HOX-mediated critical cancer-associated genes involved in the cancer hallmark processes curated from the MSigDB. **d** The architectural chromatin regulator *CTCF* was noted to be the downstream target of posterior *HOXs* in the cluster. The RNA‒protein interactions of lncRNAs with **e** homeoboxes in HOX clusters and **f** epigenetic factors involved in chromatin modifications. The direction of the cross-delta arrow represents the targeted gene, whereas the edges indicate the interaction between the two nodes, and the connecting arrows indicate the mechanistic action between the associated nodes. MSigDB—Molecular signatures database; lncRNA—long noncoding RNA

In silico analysis revealed enrichment of the DNA methylation machinery in the *HOXA9* and *HOXC12* gene promoters. Proteins involved in facultative and constitutive heterochromatin, such as CBX8 (Chromobox 8), EED (Embryonic ectoderm development), EZH2 (Enhancer of zeste 2 polycomb repressive complex 2 subunit), PHC1 (polyhomeotic homologue 1), RNF2 (Ring finger protein 2), SUZ12 (Suppressor of zeste 12 homologue), BMI1 (Polycomb ring finger), CBX2 (Chromobox 2), and SETDB1 (SET domain bifurcated histone lysine methyltransferase 1), bind to the promoters of homeobox genes in the cluster. The architectural heterochromatin-associated factor CTCF (CCCTC-binding factor), which acts as an insulator, was frequently enriched near the promoter regions of *HOX* genes and sporadically across the *HOX* cluster, defining its chromatin landscape. Chromodomain-associated CHD4 (chromodomain helicase DNA binding protein 4), SMARCA4 (SWI/SNF-related, matrix-associated, actin-dependent regulator of chromatin, subfamily a, member 4), MTA3 (metastasis associated 1 family member 3) and ARID3A (AT-rich interaction domain 3 A) were predicted to have significant binding sites on *HOX* promoters, potentially contributing to chromatin remodeling. Histone methylation and acetylation writers such as RNF2, ASH2L (ASH2-like, histone lysine methyltransferase complex subunit), EZH2, SETDB1 (SET domain bifurcated histone lysine methyltransferase 1), NSD2 (nuclear receptor binding SET domain protein 2), ATF2 (activating transcription factor 2), KAT2A (lysine acetyltransferase 2 A) predicted to have binding sites on *HOX* promoters. Histone methylation readers such as CBX3 (Chromobox 3), chromodomain-associated proteins (CHD1, 2, 4, and 7) and ING4 (inhibitor of growth family member 4) were enriched in the *HOX* gene promoter. In addition to p53 and Kruppel-like factors (KLFs), promoters of anterior *HOX* genes were noted to contain binding sites for retinoic acid response elements (RAREs) including the RAR (retinoic acid receptor) and RXR (retinoid X receptor) forms of retinoid receptors (Fig. 5a). Among the *HOX* clusters, *HOXA5* and *HOXC9* exhibited a higher number of promoter-dependent interactions with neighboring *HOX* genes. Interactions such as *HOXA4-HOXA5*, *HOXA9-HOXA10*, *HOXA10-HOXA11*, *HOXA5-HOXA9*, *HOXA5-HOXA7*, *HOXB2-HOXB5*, *HOXB5-HOXB6* and *HOXC9-HOXC10* indicate potential HOX transcription factor-mediated regulation within the cluster (Fig. 5b).

The transcription factor regulation of HOX proteins in cancer hallmarks may involves downstream interactions with *EGR3* (early growth response 3)*,* which functions in apoptosis; *EGR1* (early growth response 1) and *EGR2* (early growth response 2) in TNFA (tumor necrosis factor alpha) signaling; *MYC*, a proto-oncogene in inflammatory response pathways; *BHLHE40* (basic helix-loop-helix family member e40) in hypoxia signaling; *CCND1* (cyclin D1) in the G2M checkpoint; *HES1* (hes family bHLH transcription factor 1) in Notch regulation; and *CTCF* in chromatin remodeling. Within the *HOX* cluster, HOXB9 and HOXC10 were the only proteins predicted to be directly involved in the G2/M checkpoint and hypoxia pathways, but they may also indirectly regulate effectors of the other hallmark pathways (Fig. 5c). Furthermore, HOX proteins are predicted to centrally regulate the CTCF factor, thereby reciprocally participating in epigenetic regulation and maintenance of the chromatin architecture, in addition to the bromodomain and chromodomains (Fig. 5d).

### *HOX*-embedded lncRNAs may interact with epigenetic modifiers

HOX-embedded long noncoding RNAs (HOXlncRNAs) belonging to the *HOXC* cluster, such as *HOXC-AS1*, *HOXC-AS2*, *HOXC-AS3* and *HOXC13-AS*, were predicted to interact with posterior *HOXC* genes. *HOXD-AS1* may regulate posterior *HOXD* genes, whereas *HOTAIR* lncRNA located between *HOXC11* and *HOXC12* predicted to interact with histone 3 (H3), EP300 (E1A binding protein p300), MLL1 (lysine methyltransferase 2 A), MLL3 (lysine methyltransferase 2 C), PRC2 (Polycomb repressive complex 2), EZH2, EED, SUZ12 (SUZ12 polycomb repressive complex 2 subunit), and KDM1A (lysine demethylase 1 A). Intermediately, SMARCA4, HDAC9 (Histone deacetylase 9), and DNMT1 (DNA methyltransferase 1) may be regulated by *HOXA11-AS*. These observations suggest a potential role for HOXlncRNAs in regulating of the epigenetic machinery (Fig. 5e–f).

### NAT-mediated regulation of *HOX* clusters by HOXlncRNAs

**Fig. 6:**
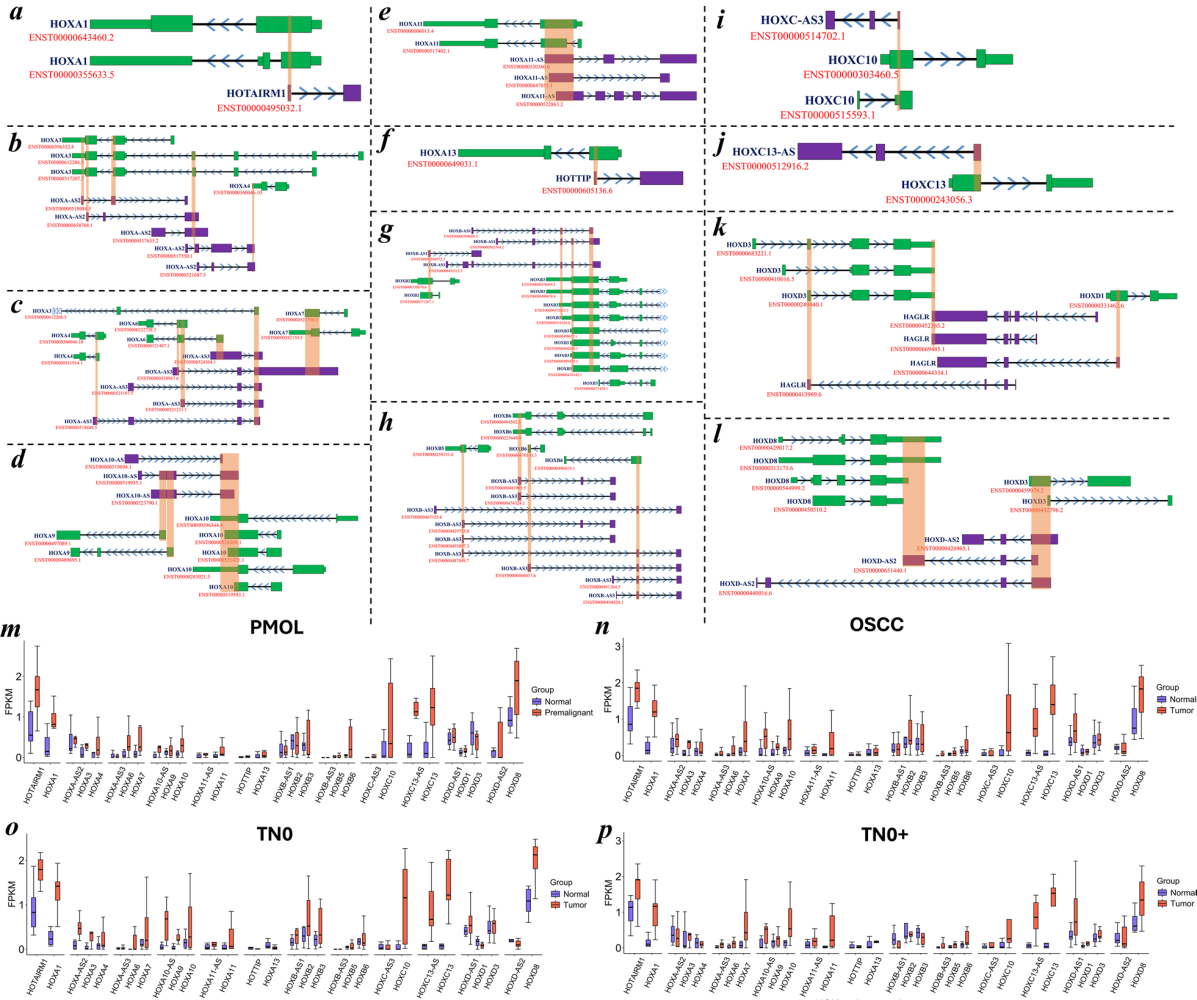
**a–p** Clustered positioning of *HOX* genes in axis with the lncRNAs located in the complementary strand. **a–l** Schematic illustration of embedded lncRNAs that act as NATs in the *HOX* cluster. *HOTAIRM1* is antisense to *HOXA1*, and *HOXA-AS2* is antisense to *HOXA3* and *HOXA4*. *HOXA-AS3* is antisense to *HOXA3*, *HOXA4*, *HOXA6*, *HOXA7*, and *HOXA10-AS* is antisense to *HOXA9* and *HOXA10*. *HOXA11-AS* is antisense to *HOXA11,* whereas *HOTTIP* is antisense to *HOXA13*. *HOXB-AS1* is antisense to *HOXB2* and *HOXB3,* while *HOXB-AS3* is antisense to *HOXB5* and *HOXB6*. *HOXC-AS3* is antisense to HOXC10, and *HOXC13-AS* is antisense to *HOXC13*. *HAGLR* is antisense to *HOXD1* and *HOXD3,* and *HOXD-AS2* is antisense to *HOXD3* and *HOXD8*. The exonic position follows the directionality arrow along the nucleotide axis defining the orientation (forwar*d* >; revers*e* <). The shaded region indicates the complementary positioning of the NATs to the *HOXs* in the cluster. **m–p** The expression of *HOX* genes with antisense sequences and embedded *HOX*-embedded lncRNAs across the *HOX* cluster was visualized as boxplots in the PMOL (*n* = 7 normal & *n* = 7 tumor), OSCC (*n* = 15 normal & *n* = 18 tumor), TN0 (*n* = 7 normal & *n* = 9 tumor) and TN0 + (*n* = 8 normal & *n* = 9 tumor) groups

Based on genomic organization, the anterior *HOXA* genes, including *HOXA1*, are positioned in complementarity to *HOTAIRM1*, whereas *HOXA3*, *HOXA4*, *HOXA6* and *HOXA7* are arranged in the antisense orientation relative to the *HOXA-AS3* lncRNA (Fig. 6a–f). *HOXB-AS1, HOXB-AS3, HAGLR* and *HOXD-AS2* antisense transcripts were positioned across in the *HOXB* and *HOXD* clusters (Fig. 6g–l). *HOX* genes and their natural antisense lncRNA pairs, such as *HOTAIRM1* and *HOXA1*, *HOXA10-AS* and *HOXA10*, *HOXC13-AS* and *HOXC13*, *HOXD-AS1* and *HOXD3* exhibited similar expression patterns, indicating that their regulation mediated post-transcriptionally through the NAT mechanism (Fig. 6m‒p). However, the heterogeneity observed in other *HOX* genes and associated antisense lncRNAs is likely due to sample-specific variations resulting from changes in the epigenetic and genomic profiles of patients. Our findings uncovered the intricate antisense-mediated regulation of *HOX* genes by embedded lncRNAs in the *HOX* cluster.

### Functional consequences of a dysregulated *HOX* network in oral cancer

**Fig. 7:**
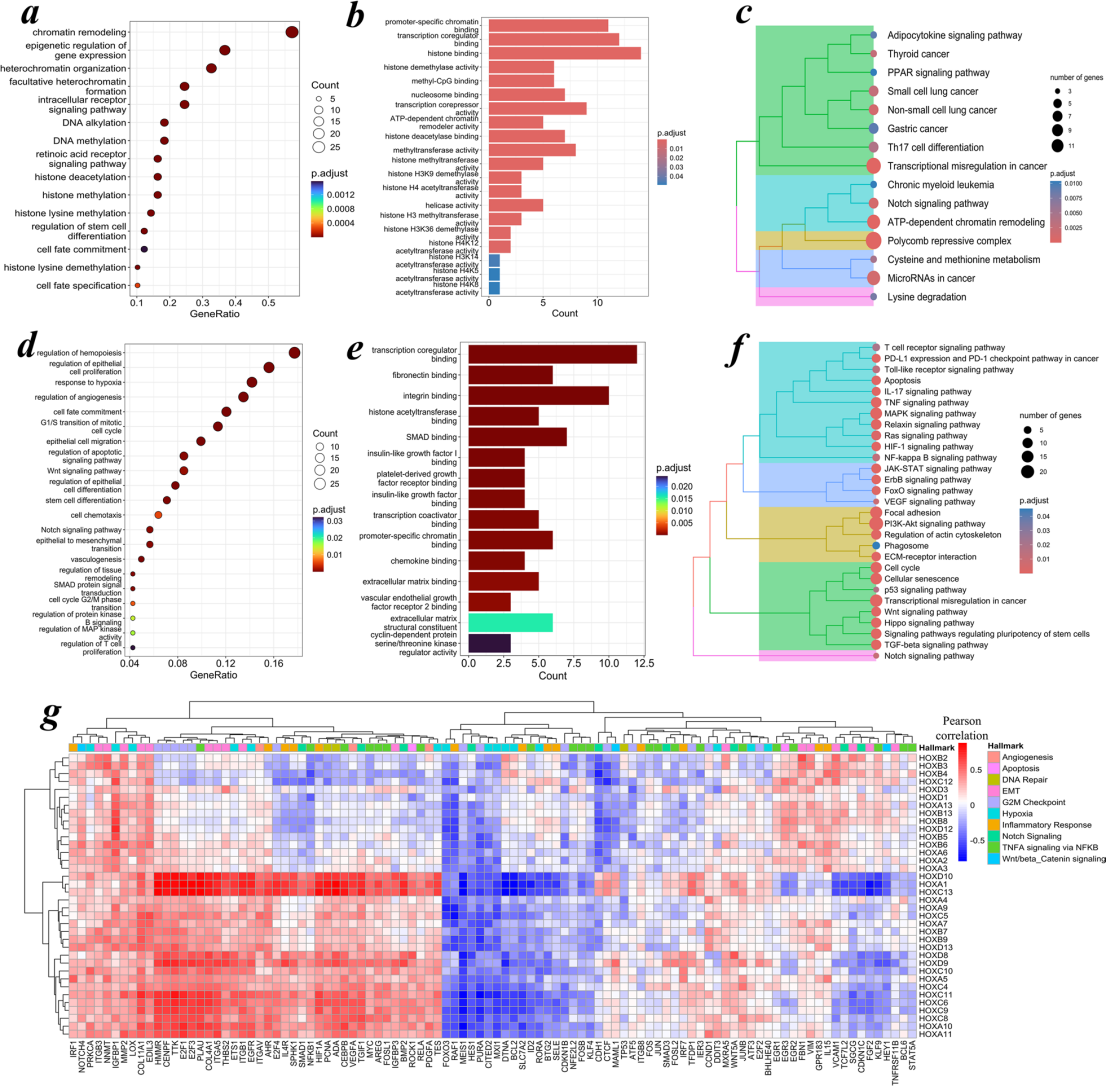
**a–g** Functional annotation of upstream and downstream targets of Homeoboxes. Functional overrepresentation analysis of the **a–c** upstream factors acting on the *HOX* genes and **d–f** downstream targets of HOX using the gene ontology (GO) biological process (BP) terms, illustrated as dot plots; molecular function (MF) terms, represented as bar plots; and hierarchically clustered KEGG pathways, based on Wald’s average distance method, visualized as tree plots. The upstream and downstream targets of HOX were significantly enriched with epigenetic and cancer signaling pathway events. An adjusted p value o*f* < 0.05 was considered statistically significant with the Bonferroni‒Hochberg correction. **g** Correlations were analyzed in *n* = 33 (15 normal and 18 tumor) patient transcriptomic gene signatures. The Pearson correlation test was applied to determine the degree of correlation between *HOX* and cancer hallmark-associated genes. A correlation coefficient of *r* > 0.3 was considered to indicate a moderate correlation, whereas *r* > 0.7 was considered to indicate a strong correlation. A p-valu*e* < 0.05 was considered to indicate statistical significance. *r* = Pearson correlation coefficient; KEGG—Kyoto Encyclopedia of Genes and Genomes

The enrichment analysis of the upstream factors acting on the *HOX* cluster revealed significant associations with DNA epigenetic regulatory functions, including chromatin organization and remodeling events (Fig. 7a–c). The downstream functional annotation of the HOX target genes revealed enrichment in various cell biological processes, such as the regulation of cell proliferation, angiogenesis, vasculature, migration, adhesion, vasculogenesis and alteration of cell-cycle checkpoints. Additionally, signaling pathways such as PD-L1 (Programmed death ligand 1), apoptosis, IL-17 (Interleukin 17), TNF, MAPK (mitogen-activated protein kinase), RAS, VEGF (vascular endothelial growth factor), PI3K-Akt, the cell cycle, p53, Wnt and NOTCH could be functionally altered by the downstream targeting of HOX proteins on critical cancer-associated genes during oral carcinogenesis (Fig. 7d–f).

Gene-to-gene correlation analysis (*r* > 0.3, *p* < 0.05) using downstream targets of *HOX* in patient transcriptomic gene signatures (*n* = 33) revealed significant correlations with the target genes associated with cancer hallmarks. The anterior *HOX* genes, particularly the hypermethylated *HOXB* cluster, showed significant negative correlations with cancer hallmark processes. The posterior *HOX* genes, predominantly those in the *HOXC* and *HOXD* clusters, were strongly positively correlated with angiogenesis, epithelial–mesenchymal transition (EMT) and G2-M checkpoint hallmarks, whereas significantly altered correlation states were observed across the inflammatory response, hypoxia and TNFA signaling-mediated pathways. Notably, *HOXA1*, *HOXC13* and *HOXD10* were strongly correlated with cancer hallmarks, indicating their regulatory role in carcinogenesis (Fig. 7e). These aberrations in disease states could result in transcriptional misregulation of HOX transcription factors, resulting in the promotive effects of cancer hallmarks during the progression of OSCC.

## Discussion

Epigenetic regulation is a complex molecular process involving the intricate interplay of DNA methylation, histone modifications, nucleosome remodeling and noncoding RNA interactions. These regulated epigenetic mechanisms are crucial for mammalian development, cellular differentiation and tissue-specific organization. When disrupted, they can lead to a loss of function that either silences or activates cancer-related genes, playing a significant role in cancer epigenetics [40]. The involvement of these developmentally regulated *HOX* genes in carcinogenesis may stem from the loss of epigenetic function [12], which directly or indirectly affects the expression of oncogenes and tumor suppressors. Intriguingly, the *HOXB3* [41] and *HOXB7* [42] genes have been implicated in epigenetic mechanisms such as DNA methylation and histone posttranslational modifications, contributing to carcinogenesis either directly or indirectly.

The dysregulation of *HOX* genes was likely due to modifications caused by altered methylation profiles across the gene, from the promoter to the gene body, which impacts regulatory dynamics. This led to an exploration of altered methylation profiles, which have been proposed as markers of diagnostic or prognostic relevance in cancer progression [43, 44]. One such observation in our study was a set of eight specific CpG sites located within the intronic region of *HOXB9,* which showed distinct patterns between potentially malignant cases and the advanced stages of tumor progression.

The transcriptionally active *HOXA3* promoter was found to be epigenetically regulated by DNA methylation in the OSCC, with promoter methylation showing an inverse correlation with gene expression [45]. Similarly, the substantial enrichment of the DNA methylation machinery in the predicted promoter of the *HOXA9* gene, as we observed, could explain the increased methylation in patients with a greater risk of metastasis in OSCC [46]. Specifically, an increase in exonic methylation was observed in *HOXA4* and *HOXD3*, whereas intronic methylation was increased in *HOXA6* and *HOXB9* in OSCC compared with PMOL. Although our study captured overall trends of the *HOX* gene methylation relative to expression, further stratification of the samples by early and advanced stages may offer additional insights into the dynamic epigenetic regulation associated with disease progression.

During development, chromatin accessibility plays a crucial role in governing *HOX* gene expression. With respect to open chromatin, enhancers are located either upstream or downstream of *HOXs,* which allows specific transcription factors to modulate gene expression [47]. The dysregulation of chromatin accessibility at *HOX* gene loci [47] underscores the importance of this regulatory mechanism in maintaining normal developmental processes. These findings emphasize the crucial function of chromatin accessibility in controlling *HOX* gene expression and its broader implications for understanding developmental cancer biology.

Gene-wide methylation analysis of *HOX* clusters revealed constitutively unmethylated CpG patterns characterized by a loss of methylation, especially those marked with CpG islands and upstream regions, regardless of the disease state [48, 49]. These patterns of *HOX* genes may influence gene regulation through chromatin accessibility and histone modifications, potentially playing a role in carcinogenesis. Our observations revealed the presence of CURs in *HOXA1*, *HOXB5*, *HOXB6*, *HOXC5* and *HOXC10*, which constitute open chromatin regions located upstream of the gene body, whereas *HOXA11*, *HOXB9* and *HOXC11* harbor regions downstream of the promoter. All of these regions were marked by an unmethylated state in both PMOLs and OSCC samples. This pattern suggests a preserved epigenetic landscape at these loci across both disease stages. Among these *HOXA1*, *HOXA11*, *HOXC5*, *HOXC10* and *HOXC11* were significantly upregulated, whereas *HOXB5*, *HOXB6* and *HOXB9* displayed heterogeneous expression in OSCC.

Additionally, histone modifications positioned in nucleosomes flanking these CURs may regulate chromatin state, thus influencing gene regulation as reported earlier [49]. The dynamic remodeling of euchromatin and heterochromatin states in these genomic regions corresponds with gene expression signatures in oral cancer and other cancer cell types [49]. These regulatory hotspots, characterized by constitutive CpG unmethylation and chromatin accessibility, are likely to serve as epigenetic scaffolds that facilitate disease-specific transcriptional regulation.

CTCF binding sites often function as insulators or boundary elements that segregate distinct chromatin domains [50], which may delineate the boundaries around *HOX* gene clusters and isolating them from neighboring genomic regions. This organization may be crucial for preserving the spatial and temporal expression of *HOX* genes during development. By forming loops and establishing interactions between regulatory elements (enhancers or silencers) and *HOX* gene promoters, CTCF could play a role in regulating the accessibility of these genes to the transcriptional machinery.

The lncRNAs embedded within the *HOX* cluster, which act through RNA‒protein interactions, represent a significant aspect of RNA-driven epigenetics [51, 52]. Furthermore, the clustered organization of *HOX* genes with embedded long noncoding RNAs reveals complementary positioning, a phenomenon termed natural antisense-mediated regulation [52, 53]. Our findings highlight the controlled regulation of the *HOXA* cluster by naturally occurring antisense lncRNA transcripts located on the opposite strand of the corresponding coding genes, consistent with the previous observation of *HOXA10-AS* mediated regulation of *HOXA10* [49]. Notably, the poor prognosis and overexpression of HOXA1 reported in OSCC [54] are likely attributed to the open chromatin nature of the promoter and the positive regulation by HOTAIRM1 through a natural antisense-mediated mechanism reported in our study.﻿

The functional consequences of *HOX* genes are likely due to their transcriptional misregulation resulting from cancer disease progression caused by the loss of their fine-tuned epigenetic landscape in the *HOX* cluster. Furthermore, analysis of differentially dysregulated *HOX* genes using knockdown and/or overexpression models, with a focus on transcription factor-mediated regulation within the *HOX* cluster, could help elucidate the transcriptional regulatory landscape of *HOX* genes in cancer progression. Characterizing their roles across cancer types, their impact on molecular gene networks, and their potential as therapeutic targets may be essential for advancing both cancer diagnosis and treatment.

## Conclusion

*Homeobox* genes exhibited differential methylation and expression across the clusters. Clinically, *HOXB9* intronic CpG sites could serve as clinically relevant diagnostic markers distinguishing leukoplakia and advanced oral cancer groups. Mechanistic alterations caused by DNA epigenetic processes, such as an open chromatin structure, DNA methylation, and RNA epigenetics mediated by *HOX*-embedded lncRNAs, may tightly regulate *HOX* gene expression. *HOXA1*, *HOXD10* and *HOXC13* are strongly correlated with cancer hallmarks. Our findings suggest the intricate interplay of DNA and RNA epigenetic mechanisms on *HOX* genes, highlighting the functional role of transcriptional misregulation in contributing to oral cancer progression through the targeting of critical cancer-associated genes.

## Supplementary Information

Additional file1

Additional file2

Additional file3

Additional file4

Additional file5

## Data Availability

The publicly archived datasets (TCGA Pan-cancer WGBS and ATAC-seq) used in this present study are freely available for the research community to access from UCSC Xena (https://xenabrowser.net/datapages/). The authors state that all data necessary for confirming the conclusions presented in this article, where applicable, are represented fully within the article or can be provided by the authors upon request.
