# Supplementary material for: Multifaceted regulation of the HOX cluster and its implications in oral cancer
Source: Clin Epigenetics. 2025 Jul 17;17:126. doi: 10.1186/s13148-025-01933-w (PMC12273044; doi:10.1186/s13148-025-01933-w)
Supplement: Supplementary file 1 — Additional file1 [file 13148_2025_1933_MOESM1_ESM.docx]

**Supplementary Figure S1**


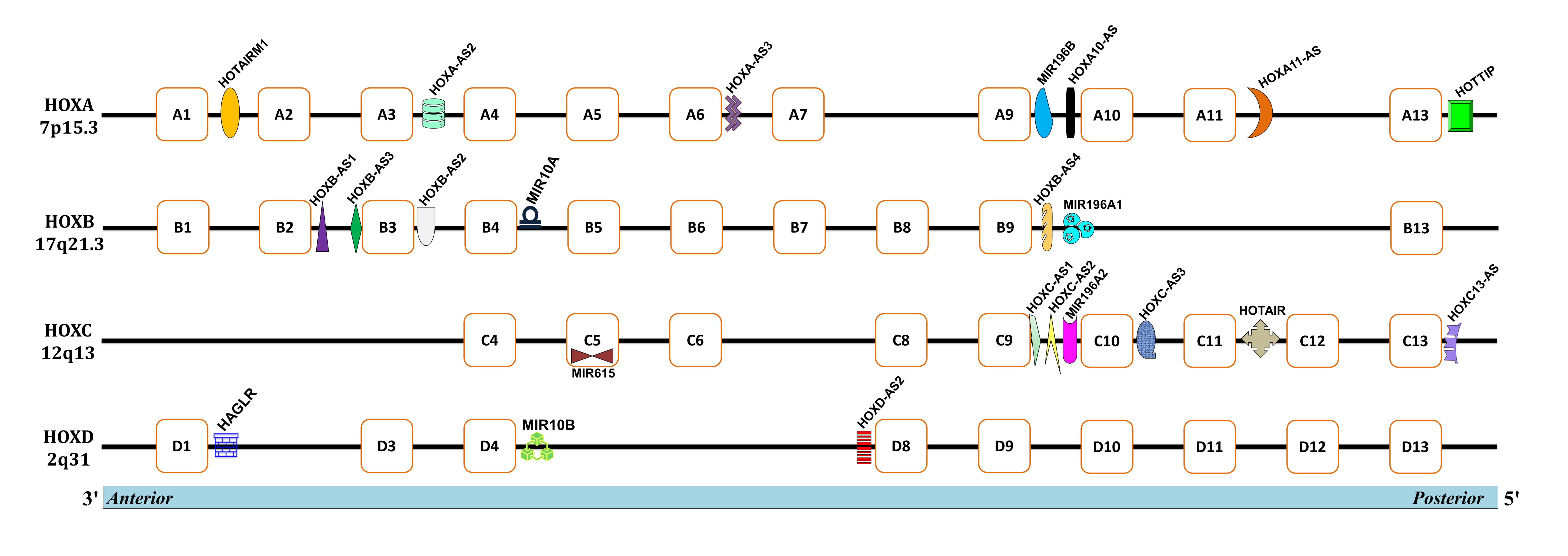


**Supplementary Figure S1:** Schematic illustration of Homeobox (*HOX*) genes and the embedded non-coding RNAs (ncRNAs) in the *HOX* cluster. In mammals, a total of 39 *HOX* genes are segregated into four clusters, namely *HOXA* (7p15.3), *HOXB* (17q21.3), *HOXC* (12q13), and *HOXD* (2q31). The *HOX* genes situated towards the 3′ are termed as the anterior *HOX* whereas those located towards the 5′ end was coined as the posterior *HOX* genes. Among the embedded ncRNAs in the cluster, 17 are long non-coding RNAs and 6 are the micro-RNAs.
