# Supplementary material for: Multifaceted regulation of the HOX cluster and its implications in oral cancer
Source: Clin Epigenetics. 2025 Jul 17;17:126. doi: 10.1186/s13148-025-01933-w (PMC12273044; doi:10.1186/s13148-025-01933-w)
Supplement: Supplementary file 2 — Additional file2 [file 13148_2025_1933_MOESM2_ESM.docx]

**Supplementary Figure S2**


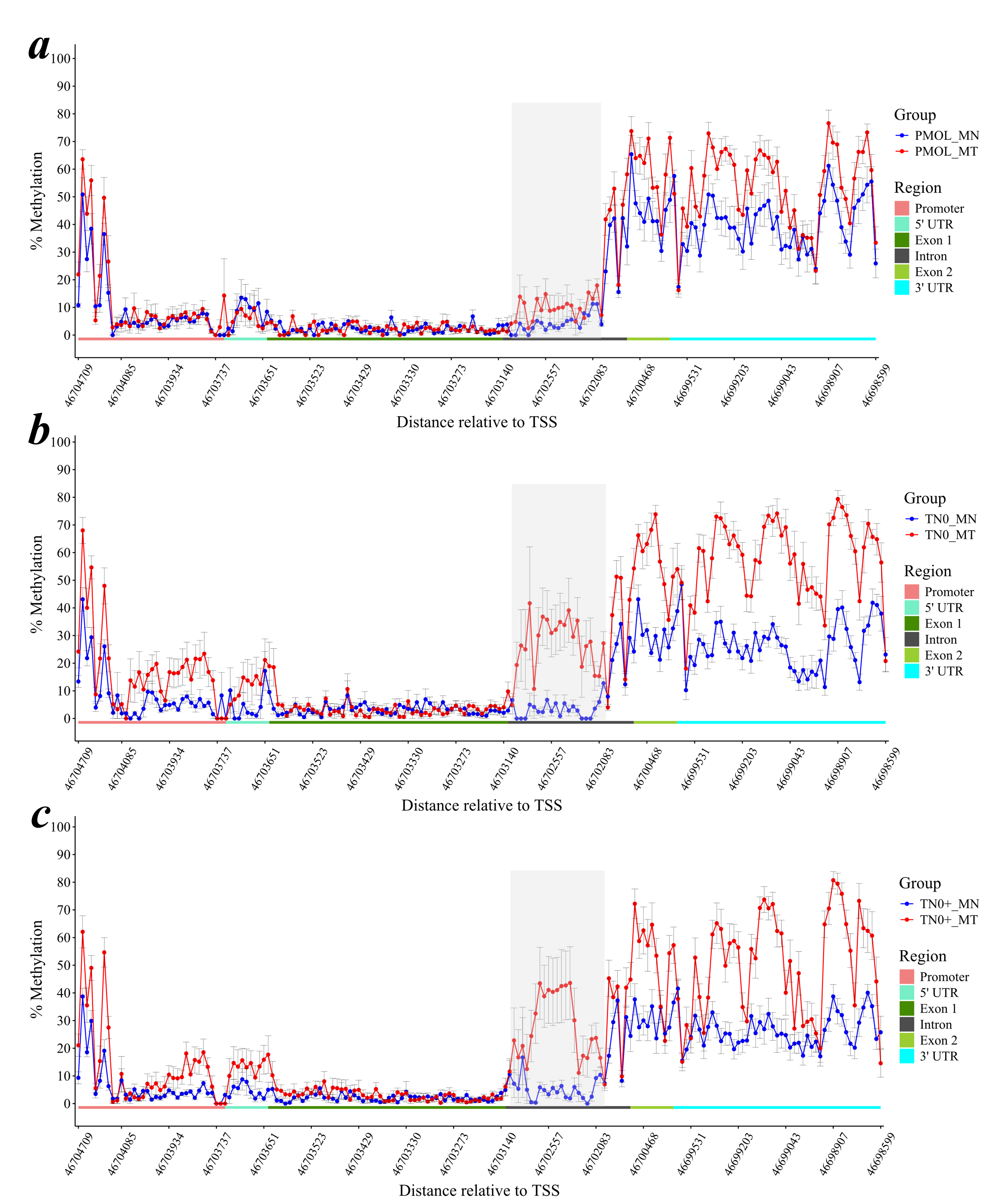


**Supplementary Figure S2:** Gene-wide methylation profile of *HOXB9* in **a)** PMOL (n=8), **b)** TN0 (n=6) and **c)** TN0+ (n=8) matched case-normal samples. The region further assayed for the ROC-AUC analysis was shaded in the gray color. Significant increase of methylation was evident in the intronic region with the increased tumor progression. ‘MN’ refers to matched normal whereas ‘MT’ refers to matched tumor, derived from the same case.
