# Supplementary material for: Multifaceted regulation of the HOX cluster and its implications in oral cancer
Source: Clin Epigenetics. 2025 Jul 17;17:126. doi: 10.1186/s13148-025-01933-w (PMC12273044; doi:10.1186/s13148-025-01933-w)
Supplement: Supplementary file 3 — Additional file3 [file 13148_2025_1933_MOESM3_ESM.docx]

**Supplementary Figure S3:**


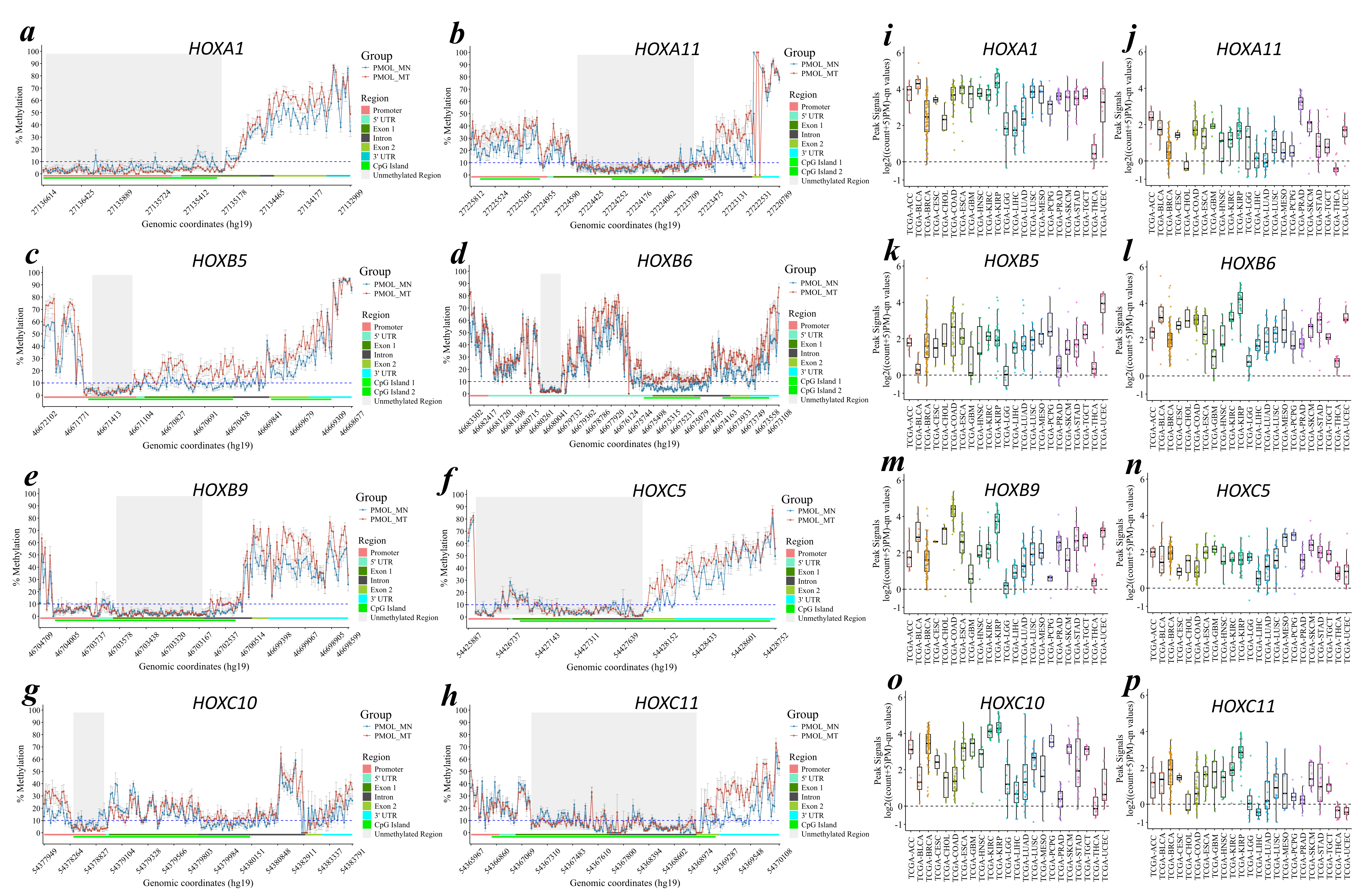


**Supplementary Figure S3 (a-p):** Locus-specific methylation and chromatin accessibility patterns at *HOX* gene clusters in oral lesions and across cancers. **(a–h)** Constitutively unmethylated regions across *HOX* genes in potentially malignant oral lesions (PMOLs) exhibited locus-specific methylation patterns consistent with those observed in OSCC samples. **(i–p)** Tumor type-specific ATAC-seq analysis across 23 TCGA cancer types—including TCGA-ACC (n=9), BLCA (n=10), BRCA (n=74), CESC (n=2), CHOL (n=5), COAD (n=38), ESCA (n=18), GBM (n=9), HNSC (n=9), KIRC (n=16), KIRP (n=34), LGG (n=13), LIHC (n=17), LUAD (n=22), LUSC (n=16), MESO (n=7), PCPG (n=9), PRAD (n=26), SKCM (n=13), STAD (n=21), TGCT (n=9), THCA (n=14), and UCEC (n=13) revealed pronounced chromatin accessibility peaks at these *HOX* loci. The consistently elevated ATAC-seq signals across multiple tumor types, including TCGA-HNSC, highlight the open chromatin configuration of these regions, supporting their potential role as regulatory hotspots. The sample or disease-specific variability observed may be attributed to altered gene expression signatures potentially influenced downstream by the chromatin remodelling.
