# Supplementary material for: Multifaceted regulation of the HOX cluster and its implications in oral cancer
Source: Clin Epigenetics. 2025 Jul 17;17:126. doi: 10.1186/s13148-025-01933-w (PMC12273044; doi:10.1186/s13148-025-01933-w)
Supplement: Supplementary file 4 — Additional file4 [file 13148_2025_1933_MOESM4_ESM.docx]

**Supplementary Figure S4**


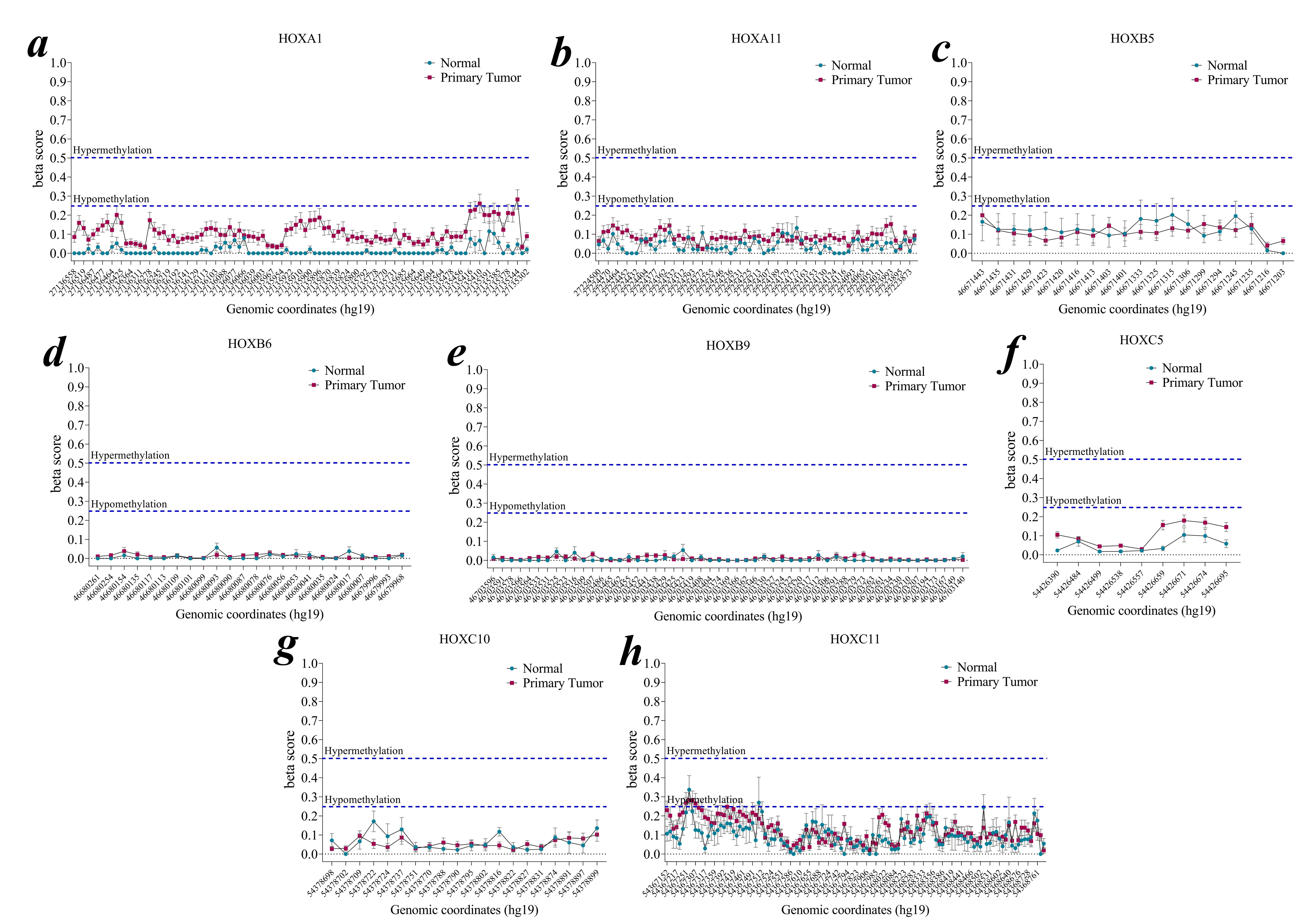


**Supplementary Figure S4:** Methylation profile of the constitutively unmethylated regions in the identified *HOX* genes. CURs analyzed in normal (n=8) and primary tumor (n=39) cases using the TCGA pan-cancer cohort. The results are in congruent with our findings observed in the clinical cohort where the marked CpG sites were hypomethylated irrespective of the sample and the cancer type. These findings shows that the identified regions exhibiting the ‘constitutive unmethylation’ marks in the *HOX* cluster were consistently observed in the other cancer types.
