# Supplementary material for: Multifaceted regulation of the HOX cluster and its implications in oral cancer
Source: Clin Epigenetics. 2025 Jul 17;17:126. doi: 10.1186/s13148-025-01933-w (PMC12273044; doi:10.1186/s13148-025-01933-w)
Supplement: Supplementary file 5 — Additional file5 [file 13148_2025_1933_MOESM5_ESM.docx]

**Supplementary Figure S5**


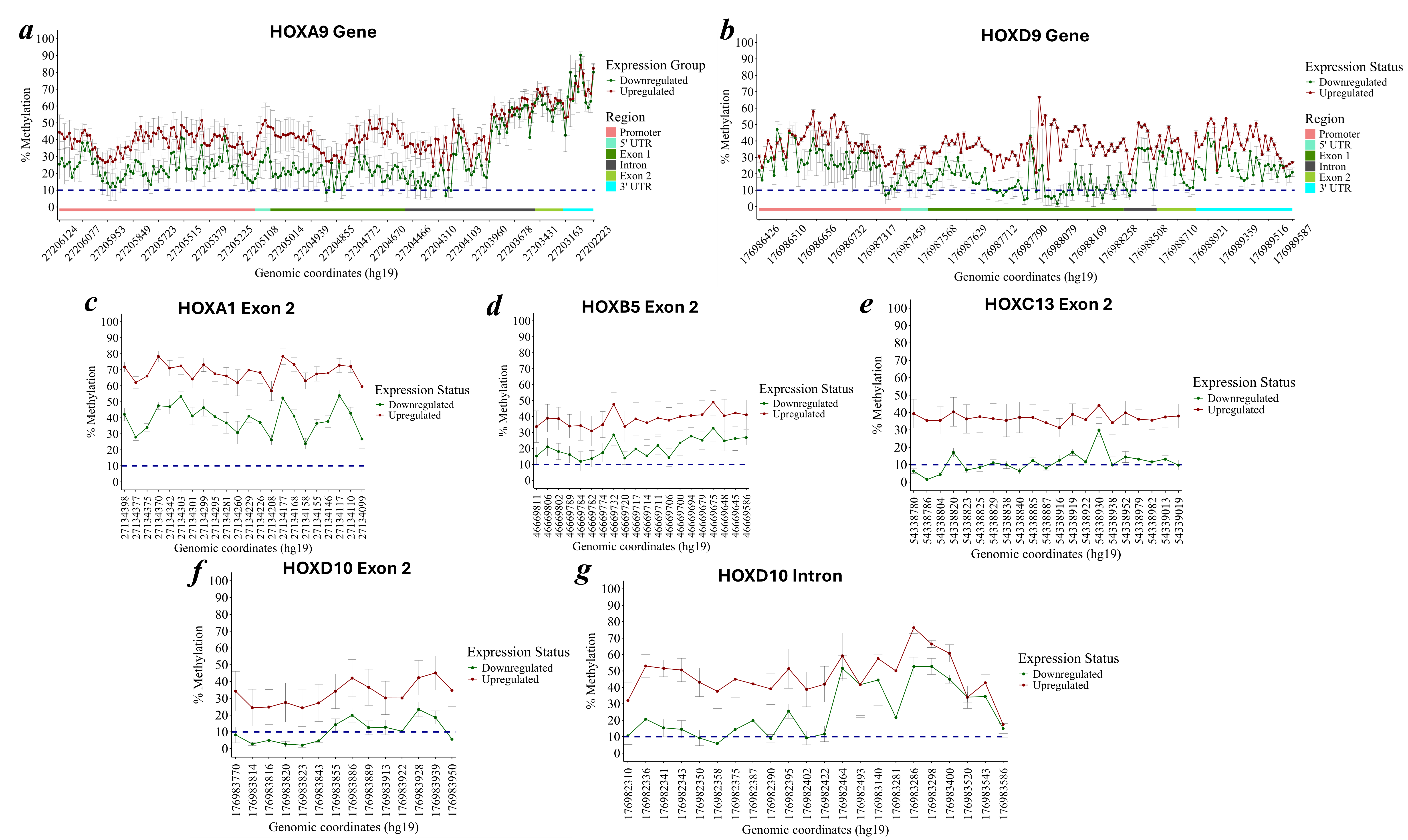


**Supplementary Figure S5:** Patterns of *HOX* gene methylation positively correlating with the gene expression in OSCC. Line plots depicting the differential methylation patterns observed in the *HOXA9, HOXD9, HOXA1, HOXB5, HOXC13* and *HOXD10* gene regions analyzed in OSCC patients (n=14) based on their expression status. A cutoff <10% (represented in dashed blue color) was unmethylated.
